# Mitochondrial Quality Control and Disease: Insights into Ischemia-Reperfusion Injury

**DOI:** 10.1007/s12035-017-0503-9

**Published:** 2017-04-11

**Authors:** Anthony R. Anzell, Rita Maizy, Karin Przyklenk, Thomas H. Sanderson

**Affiliations:** 1 Department of Emergency Medicine, Wayne State University School of Medicine, Detroit, MI 48201 USA; 2 Cardiovascular Research Institute, Wayne State University School of Medicine, Detroit, MI 48201 USA; 3 Department of Physiology, Wayne State University School of Medicine, Detroit, MI 48201 USA

**Keywords:** Brain, Mitophagy, Ischemia, Reperfusion, Mitochondria, Mitochondrial dynamics

## Abstract

Mitochondria are key regulators of cell fate during disease. They control cell survival via the production of ATP that fuels cellular processes and, conversely, cell death via the induction of apoptosis through release of pro-apoptotic factors such as cytochrome C. Therefore, it is essential to have stringent quality control mechanisms to ensure a healthy mitochondrial network. Quality control mechanisms are largely regulated by mitochondrial dynamics and mitophagy. The processes of mitochondrial fission (division) and fusion allow for damaged mitochondria to be segregated and facilitate the equilibration of mitochondrial components such as DNA, proteins, and metabolites. The process of mitophagy are responsible for the degradation and recycling of damaged mitochondria. These mitochondrial quality control mechanisms have been well studied in chronic and acute pathologies such as Parkinson’s disease, Alzheimer’s disease, stroke, and acute myocardial infarction, but less is known about how these two processes interact and contribute to specific pathophysiologic states. To date, evidence for the role of mitochondrial quality control in acute and chronic disease is divergent and suggests that mitochondrial quality control processes can serve both survival and death functions depending on the disease state. This review aims to provide a synopsis of the molecular mechanisms involved in mitochondrial quality control, to summarize our current understanding of the complex role that mitochondrial quality control plays in the progression of acute vs chronic diseases and, finally, to speculate on the possibility that targeted manipulation of mitochondrial quality control mechanisms may be exploited for the rationale design of novel therapeutic interventions.

## Introduction: Mitophagic Balance in Acute and Chronic Disease

Cardiovascular and neurologic diseases are leading causes of morbidity and mortality in the USA [1]. Cardiovascular disease can result in acute injuries sustained by both the heart and the brain in the form of acute myocardial infarction (AMI) and stroke. AMI and stroke are induced by a cessation of blood flow (ischemia), caused by blockage of one or more of the coronary or cerebral arteries that supply the heart or brain. This cessation of blood flow will subsequently lead to tissue hypoxia or anoxia and, ultimately, necrotic cell death (characterized by cellular swelling and membrane rupture due to energy failure). It is well known that although restoration of blood flow (reperfusion) is essential to salvage ischemic tissue, this can also, paradoxically, exacerbate damage from several cellular alterations including excessive reactive oxygen species (ROS) production from mitochondria [2–4]. ROS production will lead to mitochondrial damage and, ultimately, mitochondrial failure and predominantly apoptosis (typically occurring via the intrinsic or mitochondrial pathway) [5, 6]. Ultimately, cell death observed during ischemia and reperfusion in both heart and brain occurs over a broad spectrum of cell death phenotypes depending on the duration and severity of the ischemic insult. This process occurring at the level of the tissue has been termed lethal ischemia/reperfusion (I/R) injury.

In addition to acute injuries such as stroke or AMI, the incidence of chronic neuropathologies (including, for example, Parkinson’s disease (PD) and Alzheimer’s disease (AD)) are on the rise as the average lifespan continues to increase [7, 8]. PD and AD are neurodegenerative diseases that affect different parts of the brain and are typically seen in the elderly, although inherited mutations may also lead to disease in younger patients. PD is a movement disorder, and while many forms of hereditary and acquired PD have unique mechanistic causes, all result in a pathologic dysfunction and death of dopaminergic neurons. AD is a neurodegenerative disorder that affects the elderly and causes memory loss and declined cognitive function. The pathological factors of the disease consist of the presence of amyloid-β plaques and neurofibrillary tangles in the brain [9]. Although I/R injury, Parkinson’s disease, and Alzheimer’s disease differ in terms of their etiology, the literature suggests that loss of mitochondrial integrity plays a central role in each of these disease processes.

It is well established that mitochondria are key regulators of cell fate, controlling survival (via the production of ATP that fuels cellular processes) and, conversely, death (via the induction of apoptosis). Indeed, mitochondrial dysfunction has been well characterized as a precursor to cell death [10–13]. Therefore, it is essential to have stringent control mechanisms regulating the quality of mitochondria to avoid the pathologic effects of dysfunctional mitochondria on the cell.

**Fig. 1:**
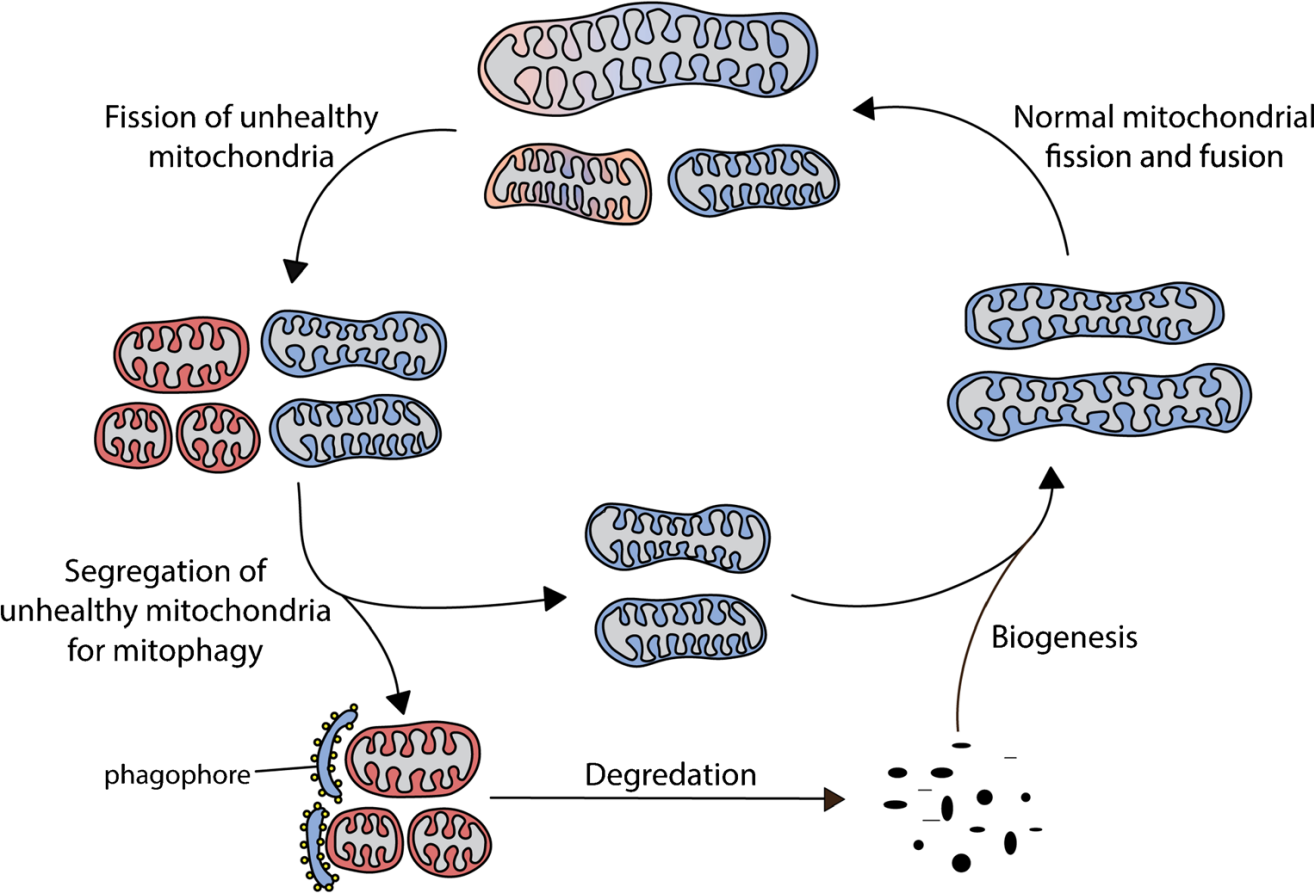
The mitochondrial quality control cycle. The mitochondrial quality control cycle involves a dynamic process of fission, fusion, mitophagy, and biogenesis. When mitochondria become depolarized or dysfunctional, they are marked for degradation. Once marked, the unhealthy component of the mitochondria will undergo fission from the healthy mitochondrial network. Certain damaged mitochondria can fuse with other healthy mitochondria in an attempt to salvage that mitochondrion, but typically, dysfunctional mitochondria will undergo mitophagy. When the dysfunctional mitochondria are segregated from the healthy mitochondrial network, mitochondria will accumulate mitophagy markers that will recruit the phagophore. The phagophore will attach to the dysfunctional mitochondria, mature into an autophagosome, fuse with the lysosome to form the autolysosome, and degrade the mitochondria. Once degraded, the cell will recycle the amino acids and fatty acids to enable the remaining healthy mitochondrial network to grow and divide through biogenesis

While the negative roles of mitochondrial failure and apoptosis are well documented, much less is known of the causal role of mitochondrial quality control in disease and the potentially nuanced role for these mechanisms in different disease settings. Importantly, evidence suggests a divergent role of mitochondrial quality control in acute vs. chronic disease. Defects in the mechanisms that regulate quality of mitochondria are recognized to play a large role in chronic diseases such as PD and AD, but, to date, their role in the acute setting of I/R injury is poorly understood. In contrast, evidence supports a salutary role for mitochondrial quality control in acute cardiac and neurologic injury, suggesting that quality control mechanisms can serve both survival and death functions depending on the nature of the disease. This review aims to (i) discuss the molecular mechanisms involved in mitochondrial quality control including mitochondrial dynamics and mitophagy (Fig. 1), (ii) detail the role that mitochondrial quality control plays in chronic and acute neurodegenerative and cardiovascular diseases, and (iii) provide a better understanding of the intricacies and balance of this process in the progression of acute vs chronic diseases.

## The Axes of Mitochondrial Quality Control

### Mitochondrial Dynamics: to Divide or Not to Divide?

Within the cell, mitochondria exist in an ever-changing dynamic state, where mitochondrial networks are constantly elongating and dividing (i.e., mitochondrial fusion and fission, respectively). The balance of these two events provides an equilibrium of small fragmented mitochondria and long interconnected mitochondrial networks and is thought to be essential for mitochondrial homeostasis, cell stability, and cell survival (Fig. 1) [14, 15]. Fission plays a role in segregating dysfunctional mitochondria that contain damaged proteins, destabilized membranes, and mutated or damaged mitochondrial DNA (mtDNA) [16–20]. Fusion, in contrast, has been shown to aid in equilibration of matrix metabolites, intact mtDNA, and even membrane components such as complex I of the electron transport chain [16, 21–24]. Fission and fusion are both regulated by a family of dynamin-related proteins (DRPs). These proteins are unique in that they are large self-assembling GTPases that also possess the capability of assembly-stimulated GTP hydrolysis [25]. Through the work of DRPs, the mitochondrial network can be in constant communication to ensure a healthy connected network, while at the same time allowing the distribution of mitochondria to specific sites of the cell via transport on actin or microtubule networks [26, 27].

#### Mitochondrial Fission

**Fig. 2:**
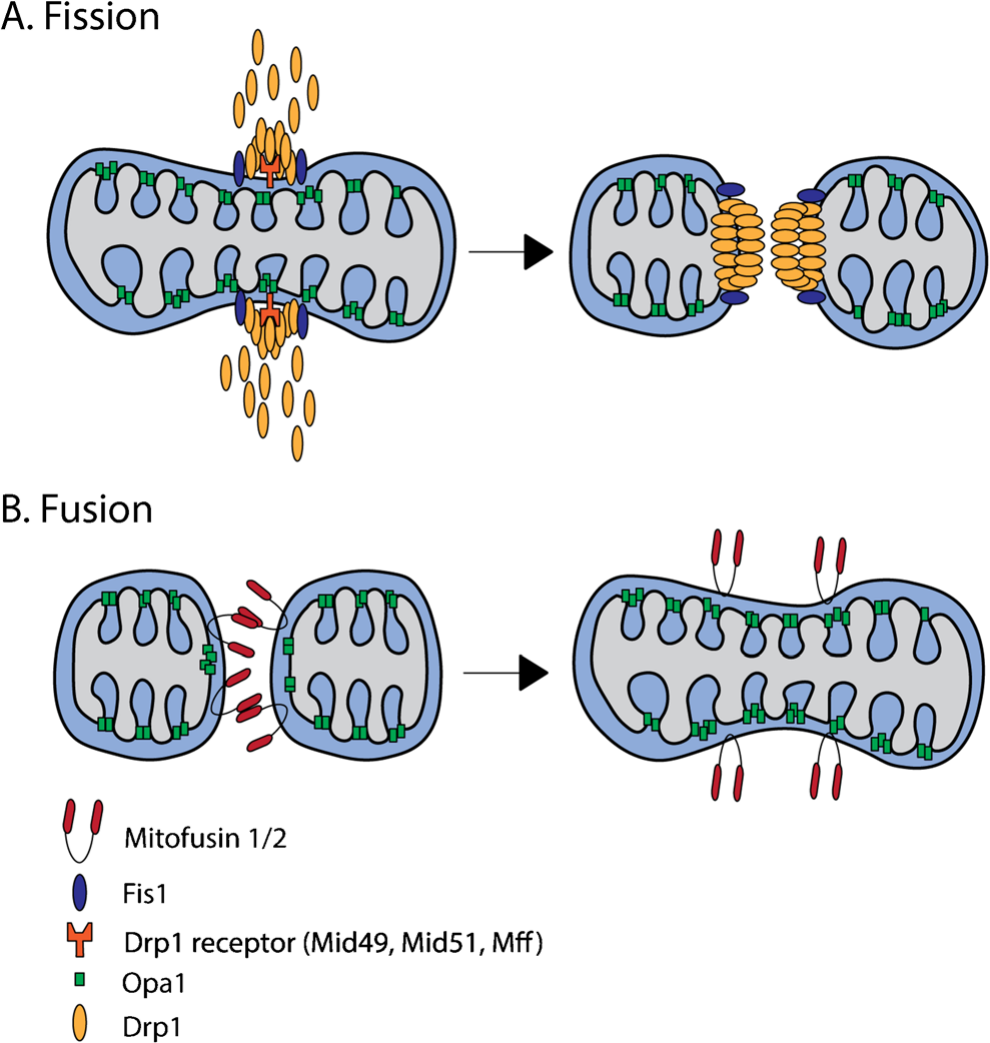
Mitochondrial dynamics. **a** Fission is mediated by a family of dynamin-related proteins (Drp). Activated Drp1 translocates from the cytosol to the mitochondrial membrane where it interacts with Drp1 receptors (Mid 49, Mid51, Mff) and Fis1 to create the fission complex. Drp1 oligomers constrict and divide the mitochondria. **b** Fusion is mediated through the mitofusins (Mfn1/2) and optic atrophy 1 (Opa1). The mitofusins mediate the fusion of the outer mitochondrial membrane, while Opa1 is thought to mediate fusion of the inner mitochondrial membrane. Mitofusins are anchored to the outer mitochondrial membrane and interact with each other and form a hemifusion stalk. The stalk then grows into a lipidic hole and finally reestablishes membrane continuity. Opa1 forms a fusion pore for the inner mitochondrial membrane via its cardiolipin binding domain

The master mediator of fission is dynamin-related protein 1 (Drp1), which has been shown to be essential for noncytokinetic mitochondrial division [16, 28]. Drp1 is distributed diffusely throughout the cytosol and, when activated through post-translational modifications (predominantly phosphorylation/dephosphorylation), translocates to the outer mitochondrial membrane via actin and microtubule mechanisms [29–33]. These post-translational modifications, described in detail below, include phosphorylation/dephosphorylation, ubiquitination, and sumoylation, in a cell-specific manner [34, 35]. Once positioned on the outer mitochondrial membrane, Drp1 interacts with four mitochondrial-bound proteins that serve as Drp1 receptors (mitochondrial dynamic proteins of 49 and 51 kDa (Mid49 and Mid51), mitochondrial fission protein 1 (Fis1), and mitochondrial fission factor (Mff), where it constricts and cleaves the mitochondria (Fig. 2a) [36–38]. Fis1 is an 18-kDa adaptor protein anchored to the outer mitochondrial membrane and has been implicated in recruiting Drp1, as well as modulating the assembly of the fission complex [39, 40]. Fis1 is thought to be required for mitochondrial fission, although this remains controversial as other groups have found it to be dispensable in the fission process [38, 41, 42].

Fission is regulated by numerous post-translational modifications of Drp1 as well as endoplasmic reticulum (ER)-mitochondrial contact sites. Phosphorylation/dephosphorylation is one of the main regulators of Drp1 and is carried out at two different serine sites located 20 amino acids apart (Ser616 and Ser637) [43]. Phosphorylation of Ser616 is associated with Drp1 activation and is phosphorylated by cyclin-dependent kinase 1 (CDK1), extracellular signal-regulated kinase (ERK1/2), and protein kinase C delta (PKCδ) [44–46]. CDK1 induces mitochondrial fragmentation during mitosis [44]. ERK1/2 and PKCδ induces Drp1-mediated mitochondrial fission via increases in ROS production during hyperglycemic conditions and hypertensive neuroencephalopathy, respectively [45, 46]. Ser637 is phosphorylated by protein kinase A (PKA), calcium/calmodulin-dependent protein kinase 1 alpha (CAMKIα), and the Rho-associated coil-containing protein kinase 1 (ROCK1) [34, 35, 47]. PKA-phosphorylated Drp1 has been shown to have decreased GTPase activity and result in decreased fission during starvation, stress, or exercise. Studies in neurons and cardiac tissue exposed to oxygen-glucose deprivation (OGD) and ischemia-reperfusion, respectively, demonstrate calcineurin-mediated dephosphorylation of Ser637, subsequently leading to Drp1 activation, mitochondrial fission, and apoptosis [33, 48, 49]. Conversely, CAMKIα phosphorylation of Ser637 results in enhanced fission during conditions of high extracellular K^+^ (inducing Ca^2+^ influx) in primary rat hippocampal neurons [33, 35, 47]. Additionally, ROCK1 phosphorylation of Ser637 has been demonstrated to induce mitochondrial fission in podocytes and endothelial cells of mice with metabolic syndrome and diabetes [34]. Phosphorylation of the same residue leading to opposite effects points to the complexity of Drp1 regulation that is likely dependent on cell type, extracellular conditions, as well as intracellular status.

Recent literature also suggests a role for the ER in mitochondrial fission. Studies conducted in both yeast and mammalian cells have shown that ER tubules will wrap around the mitochondria and mediate constriction before Drp1 recruitment via the ER-localized inverted formin 2 (INF2) mechanisms [50, 51]. INF2 is thought to drive initial mitochondrial constriction that provides sites for subsequent Drp1 recruitment and secondary constriction [51]. The multiple mechanisms involved in the regulation of fission underscore the complexity of this process and may provide insight into potential mechanisms by which dysregulated mitochondrial dynamics may interact with disease processes.

#### Mitochondrial Fusion

Fusion is mediated by three different GTPases: optic atrophy 1 (Opa1), mitofusin 1 (Mfn1), and mitofusin 2 (Mfn2) [24]. Both Mfn1 and Mfn2 mediate fusion of the outer mitochondrial membranes, while Opa1 mediates the fusion of the inner mitochondrial membrane, along with its role in maintaining normal inner membrane cristae structure [52–54]. Mitofusins, which are required for fusion, are anchored to the outer mitochondrial membrane where they interact and form a hemifusion stalk to initiate the joining of two mitochondrial membranes [16, 55]. The stalk then grows and creates a lipidic hole as well as a hemifusion diaphragm to reestablish membrane continuity. Finally, a fusion pore is made for inner membrane fusion via the lipid binding domain in Opa1 that is specific for cardiolipin [55, 56] (Fig. 2b).

Fusion of the inner and outer mitochondrial membrane is mediated mainly through proteolytic cleavage and ubiquitination, respectively. Opa1, in mammals, consists of eight different isoforms generated by alternative splicing of three of the 30 Opa1 exons [57, 58]. Membrane-bound long (L)-Opa1 can be further processed via two proteolytic cleavage sites (S1 and S2), generating short (S)-Opa1 forms [54]. Proteolytic processing is carried out predominantly through two intermembrane space AAA proteases (ATPases associated with diverse cellular activities): (i) overlapping with m-AAA (OMA1) cleaving at the S1 site and (ii) yeast mitochondrial DNA escape 1-like (YME1L) cleaving at the S2 site [59, 60]. Every (L)-Opa1isoform contains a S1 cleavage site, while about half of the (L)-Opa1 isoforms contain both S1 and S2 cleavage sites [61, 62]. Under normal physiological conditions, S1 and S2 are constitutively cleaved to produce a 50/50 ratio of (L)-Opa1 and (S)-Opa1. The balance in Opa1 isoforms is thought to mediate the balance between mitochondrial fission and fusion [63]. Under pathophysiologic conditions, such as membrane depolarization, low levels of ATP, or dysfunctional quality control mechanisms, the balance is tipped and the remaining (L)-Opa1 are cleaved by Oma1 resulting in mitochondrial fragmentation [64–68]. Mitofusins are regulated mainly by ubiquitin-mediated degradation, specifically through the PTEN-induced kinase (PINK1) and Parkin-mediated ubiquitination pathway during mitophagy [69]. This pathway will be discussed later in further detail. Abnormalities in proteolytic cleavage of Opa1 or ubiquitination of the mitofusins result in impaired fusion, changes in cristae architecture, and favor a fragmented mitochondrial phenotype.

### Mitophagy: Out with the Old, In with the New

**Fig. 3:**
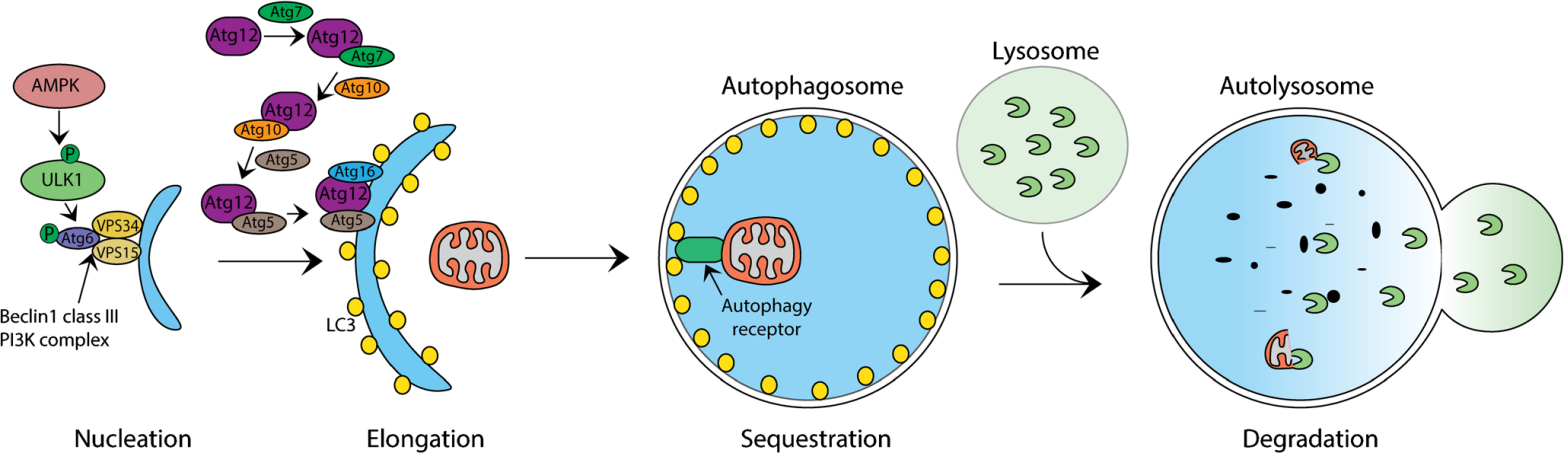
**Phases of Autophagy.** Autophagy is carried out in four different phases: nucleation, elongation, sequestration, and degradation. *Nucleation* of the isolation membrane is initiated by the phosphorylation of the ULk1 complex by AMPK. ULK1 will then recruit several autophagosome-related proteins for nucleation of the phagophore via phosphorylation of beclin 1 in the PI3K complex. *Elongation* or the extension of the autophagosome membrane is mediated by two ubiquitin-like systems involving the transferring of Atg12 from Atg7 to Atg10 and finally Atg5 where it forms a dimeric complex with Atg16 on the phagophore membrane. LC3 is also thought to help mediate the extension of the phagophore. *Sequestration* occurs when damaged organelles are detected via LC3/autophagy receptor interactions. Autophagy receptors localized on damaged organelles will bind LC3 inducing elongation of the phagophore until the cargo is completely engulfed and matures into an autophagosome. In the *degradation* phase, the lysosome will fuse with the autophagosome (autolysosome), releasing acid hydrolase enzymes that degrade the contents

Autophagy is the catabolic process of cellular components including cytosolic protein aggregates and organelles such as mitochondria that are sequestered in a double-membrane structure called an autophagosome [70, 71]. There are three distinct subtypes of autophagy [72]. *Macroautophagy* (typically referred to as autophagy) is the process of taking damaged proteins and organelles from the cytoplasm to the lysosome for degradation via an intermediate vesicle termed the autophagosome (summarized in Fig. 3). Macroautophagy typically involves the degradation of large cellular components such as organelles through both selective and nonselective mechanisms. In contrast, in so-called *microautophagy*, particles are directly taken up by the lysosome (no intermediate vesicle) by direct engulfment where they are degraded. Lastly, *chaperone-mediated autophagy* involves targeting dysfunctional proteins to be taken across the lysosomal membrane with the aid of the cytosolic chaperone heat shock cognate 70 kDa protein (HSC-70). The protein-chaperone protein complex then interacts with a specific lysosomal membrane receptor, lysosomal-associated membrane protein 2A (LAMP-2A), resulting in their degradation [73, 74].

**Fig. 4:**
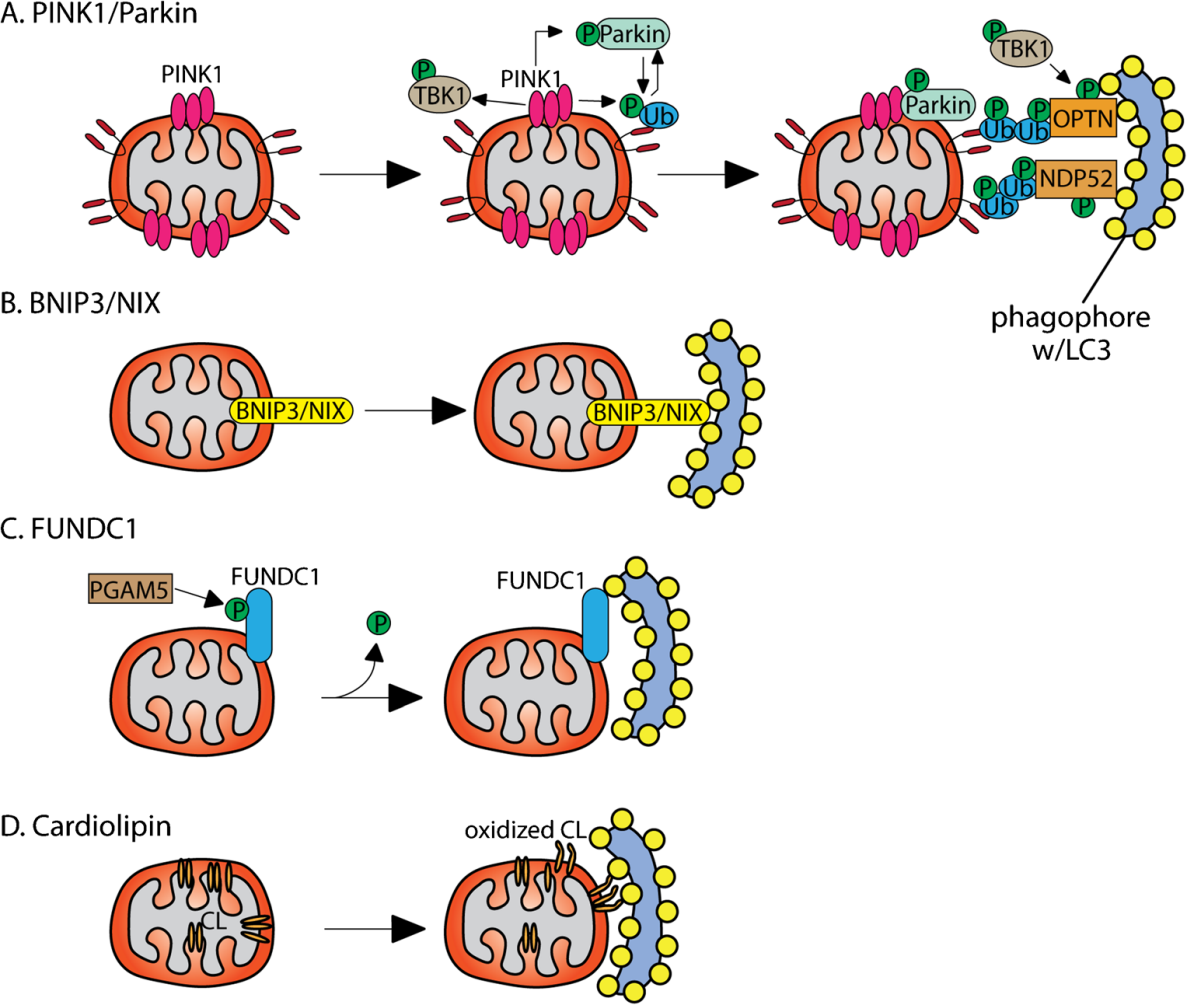
Mitophagic pathways. **a** In dysfunctional mitochondria, PINK1 accumulates in the outer mitochondrial membrane. Accumulations of PINK1 induce a concerted signaling cascade involving the simultaneous recruitment and phosphorylation of the E3 ubiquitin ligase Parkin, ubiquitin, and TBK1. Phosphorylation by PINK1 as well as phospho-Ser65-ubiquitin activates Parkin and leads to ubiquitination of outer mitochondrial membrane proteins in a feed forward process. Phagophore recruitment and binding are then mediated by OPTN and NDP52 with its ubiquitin and LC3 binding domains. **b** BNIP3/Nix are localized on the outer mitochondrial membrane and serve as mitophagy receptors and bind directly to the phagosome via LC3. **c** FUNDC1 localizes on the outer mitochondrial membrane and acts as a receptor for mitophagy under hypoxic condition. During hypoxia, PGAM5 dephosphorylates FUNDC1 and activates mitophagy via LC3 binding on the phagophore. **d** Cardiolipin localizes mainly in the inner leaflet of the inner mitochondrial membrane, specifically around the folds of the cristae. When cardiolipin is oxidized, it is externalized to the outer mitochondrial membrane where it is recognized by LC3 of the phagophore

The process of mitochondrial degradation through macroautophagy has been termed *mitophagy*. Mitophagy occurs through several different pathways (summarized in Fig. 4) that all involve (i) detection of dysfunctional mitochondria, (ii) segregation from the healthy mitochondrial network, (iii) recruitment of the phagophore, and (iv) degradation through autophagic processes. Mitophagy, in concert with mitochondrial biogenesis, ensures a healthy mitochondrial network through mitochondrial turnover. Clearance of dysfunctional mitochondria is critical to limit cellular damage via ROS production and subsequent apoptosis. Mitophagic proteins, specifically Parkin, are critical for eliminating mitochondria with deleterious mtDNA mutations via mitophagy [20]. This suggests that mitophagy is selective and plays pivotal role in the maintaining a functional population of mitochondria.

#### Autophagy: the Four Phases

There are four phases in the development of an autophagosome: (i) nucleation, (ii) elongation, (iii) sequestration and maturation, followed by (iv) fusion and degradation (Fig. 3) [75, 76].

*Nucleation* of the isolation membrane is initiated first through the phosphorylation of the Unc-51 like autophagy activating kinase 1 (ULK1) complex, typically by 5′ AMP-activated protein kinase (AMPK) [77]. Once phosphorylated, the ULK1 complex will recruit several different autophagosome-related proteins (Atg) to the autophagosome formation site. In addition, ULK1 also phosphorylates beclin 1 (Atg6) which, in turn, initiates the activity of the class III phosphotidylinositol 3-kinase (PI3K) complex (beclin 1 and vacuolar proteins sorting 34 and 15 (VPS34 and VPS15)) for nucleation of the phagophore [77–79].

*Elongation* of the phagophore is mediated by two ubiquitin-like systems (ULS) [77]. In the Atg5-Atg12 conjugation system (first of the two ULS), Atg12 is activated by Atg7 (an E1-like enzyme) and is then transferred to Atg10 (an E2-like enzyme) that is on the target protein Atg5. This Atg5-Atg12 will form a dimeric complex with Atg16, which will target the phagophore membrane. The second ULS involves the cleavage of Atg8 by Atg4 (cysteine protease) and, subsequently, the processing by the ubiquitin-like enzymes Atg7 and Atg3 [77]. Together, these ULS, along with light chain 3 (LC3), extend the autophagosome membrane.

*Sequestration* is the process by which the isolation membrane encircles the damaged organelle. This is mediated through the binding of LC3 to a variety of different proteins/receptors that detect damaged organelles [78, 80, 81]. LC3 is processed by a cysteine protease to its cytosolic form, LC3I. Cytosolic LC3I then conjugates with phosphotidylethanolamine (PE) associated with the inner and outer membrane of the phagophore to form LC3II [82]. The phagophore will then continue to elongate until it completely engulfs its cargo and matures into an autophagosome.

In the *degradation* phase, the autophagosome then fuses with the lysosome, resulting in the degradation of its cargo via acid hydrolase enzymes [75, 76]. In the fusion process, soluble NSF attachment protein receptor (SNARE) proteins, endosomal coating proteins (COPs), the endosomal sorting complex require for transport (ESCRT III) complex, the homotypic fusion and protein sorting (HOPS) complex, LAMP proteins, GTPase Rab proteins, the beclin 1 binding protein Rubicon, and chaperon HSP70 family proteins have all been implicated to play contributing roles [83–90]. More specifically, Chen et al. reported that tectonin beta-propeller repeat-containing protein 1 (TECPR1) of the lysosome binds phosphotidylinositol 3-phosphate upon conjugation of Atg12-Atg5 to promote autophagosome-lysosome fusion [91]. After degradation, the cargo’s amino acids and lipids can then be reused for synthesis of new organelles.

#### Identification of Dysfunctional Mitochondria: the Pathways of Mitophagy

Four pathways have been identified that detect dysfunctional mitochondria and recruit autophagosomes for degradation (summarized in Fig. 4). The most well-known pathway is *PINK1/Parkin-mediated mitophagy* [92, 93], named for its role in the pathogenesis of Parkinson’s disease. PINK1 contains a mitochondrial targeting domain such that, in healthy mitochondria, it is (i) transported into the intermembrane space (IMS) through the translocase of the outer mitochondrial membrane (TOM), then (ii) integrated into the inner mitochondrial membrane via insertion into the translocase of the inner mitochondrial membrane (TIM) [94], and (iii) rapidly processed and degraded by the mitochondrial membrane peptidase and presinilin-associated rhomboid-like protease (PARL). Under healthy conditions, this rapid degradation serves to keep the mitochondrial concentration of PINK1 low [95]. However, TIM-mediated import of protein relies on a steady mitochondrial membrane potential. When mitochondria are depolarized, PINK1 can no longer be inserted into the mitochondria, inhibiting its proteolytic cleavage and subsequent degradation [96]. PINK1 then accumulates on depolarized mitochondria, where it phosphorylates and activates a myriad of proteins including Parkin, ubiquitin, and TANK binding kinase 1 (TBK1) [92, 97–101] (Fig. 4a).

Parkin is an E3 ubiquitin ligase that is activated by phosphorylation by PINK at Ser65 within its ubiquitin like domain [102, 103]. In addition, Parkin has also been reported to be activated by PINK1-dependent phosphorylation of ubiquitin at Ser65 [99, 101, 104]. When activated, Parkin will ubiquitinate numerous outer mitochondrial membrane proteins including mitofusins and voltage-dependent anion channel (VDAC) [69, 105, 106]. Interestingly, the ubiquitin chains generated by Parkin are major targets of PINK1 phosphorylation, allowing Parkin retention on mitochondria, providing a feed forward mechanism to promote mitophagy [98, 104]. Concurrently, PINK1 also phosphorylates TBK1 at Ser172, promoting the phosphorylation of three different autophagy adaptor proteins: p62 (also known as sequestrome 1 (SQSTM1)), optineurin (OPTN), and nuclear dot protein (NDP52) [107–109]. The aggregation of dysfunctional mitochondria is mediated via p62, while OPTN and NDP52 serve as receptors for the phagophore via ubiquitin and LC3 binding domains [108–113] (Fig. 4a). Histone deacetylase 6 (HDAC6) will also translocate upon ubiquitination of outer mitochondrial membrane proteins and has been shown to enhance fusion of the autophagosome and lysosome [114, 115].

Mitophagy can also be regulated in a receptor-mediated fashion. One of these pathways is through *BNIP3/NIX*, which are B cell CLL/lymphoma 2 (BCL-2)-related proteins. These proteins play a dual role by both (i) inducing mitochondrial apoptosis and (ii) localizing to the mitochondrial membrane and acting as autophagy receptors where they can directly bind to LC3 [116–120] (Fig. 4b). This pathway is distinct from the PINK1/Parkin-mediated mitophagy in that PINK1/Parkin requires depolarized mitochondria to initiate mitophagy, whereas BNIP3 can activate mitophagy in mitochondria that have a stable membrane potential [92, 93, 121]. BNIP3 does recruit Parkin to mitochondria, and it has been shown that Parkin-deficient myocytes display a reduction in mitophagy despite overexpression of BNIP3 [122]. Although these proteins have dual function in activating mitophagy or inducing apoptosis, it is unclear how they are recruited for each divergent role.

FUN14 domain containing 1 (*FUNDC1*) is an outer mitochondrial membrane protein that mediates mitophagy through receptor binding with LC3 and has been implicated to play a role in hypoxia-mediated mitophagy [123] (Fig. 4c). FUNDC1 is regulated by casein kinase 2 (CK2) and the mitochondrial serine/threonine protein phosphatase PGAM5 [124]: Specifically, CK2 phosphorylates FUNDC1 to inhibit its function, while during hypoxia, PGAM5 phosphatase dephosphorylates FUNDC1 to activate its binding to LC3 and thus promote mitophagy. This pathway has been shown to be related to both PINK1/Parkin and BNIP3 primarily through PGAM5. PGAM5 phosphatase activity is required for PINK1 stabilization as well as PINK1/Parkin-mediated mitophagy, and PGAM5-deficient mice develop Parkinson’s disease [125]. BNIP3 has also been shown to be activated in hypoxia and induce mitophagy [126]. Whether or not these three pathways communicate with one another is still a question that needs further investigation.

*Cardiolipin* is a lipid predominantly localized to the inner mitochondrial membrane, is involved in mitochondrial metabolism, and interestingly, has also recently been implicated in receptor-mediated mitophagy [127, 128]. When oxidized, cardiolipin undergoes redistribution and externalization to the surface of damaged mitochondria where it is recognized by LC3 [128] (Fig. 4d). Nucleoside diphosphate kinase-D (NDPK-D (NM23-H4)), a hexameric intermembrane space protein, mediates the externalization of cardiolipin in artificially depolarized mitochondria [129]. How this process may interact with PINK1/Parkin pathway is still unknown but may provide novel insight to a potential role for cardiolipin signaling in pathologies involving mitochondrial membrane depolarization, i.e., I/R injury.

#### Mitochondrial Biogenesis

Mitochondrial biogenesis refers to the growth and division of pre-existing mitochondria. After mitochondria are degraded, the existing mitochondrial pool needs to continue growing to keep pace with energy demands of the cell. The increase in mitochondrial content involves an array of processes that include protein and lipid synthesis driven by both nuclear and mtDNA transcription. The double-stranded circular mtDNA is about 16.5 kb in length and contains 37 genes that encode for 13 proteins (subunits of electron transport chain complexes), 22 transfer RNAs, and 2 ribosomal RNAs necessary for translation [130]. Similarly, lipids such as phosphotidylethanolamine, phosphotidylglycerine, and cardiolipin are synthesized within the mitochondria from ER-derived phospholipids [131]. The rest of the ∼1000 proteins and lipids come from the nucleus and ER, respectively. This coordinated import and synthesis of proteins and lipids are essential for healthy mitochondrial biogenesis.

Peroxisome proliferator-activated receptor coactivator (PGC-1α) is considered to be the master regulator of mitochondrial biogenesis [132]. PGC-1α is induced under conditions of increased energy demand such as fasting, cold, and exercise where it increases the expression of, and coactivates, a variety of transcription factors [132–135]. These transcription factors include the nuclear respiratory factors (NRF1/2), peroxisome proliferator-activated receptor (PPAR), as well as estrogen-related receptors (ERRs) [132]. NRF1 and NRF2 promote the expression of the nuclear encoded mitochondrial transcription factor A (Tfam), which is responsible for the transcription of mtDNA [136]. As described previously, mtDNA gives rise to 13 subunits of the electron transport chain as well as the 22 tRNAs and 2 rRNAs. The nuclear proteins come from the transcriptional activity of PPARs and ERRs, which are involved in regulating the expression of proteins and enzymes that control multiple aspects of mitochondrial oxidative metabolism ranging from fatty acid transport and oxidation, glucose utilization, the TCA cycle, to oxidative phosphorylation [137]. Once transcribed in the nucleus, the mRNA is then translated in the cytosol complete with a mitochondrial localization signal. The proteins are subsequently transported in an unfolded fashion with the aid of molecular chaperones such as Hsp70 and inserted into the mitochondria through different protein translocases, including TOM and TIM (both involved in the translocation of PINK1), as well as presequence translocase-associated motor (PAM) and sorting and assembly machinery (SAM) [138].

Lipids, on the other hand, are primarily synthesized in the ER and transported to the mitochondria during biogenesis [139]. The transfer of primarily phospholipids [140] from the ER to mitochondria has been thought to be mediated via ER-mitochondrial contacts, effectively termed the mitochondria-associated membranes (MAMs) [141]. The MAMs are purportedly comprised of a variety of proteins including (i) the IP3 receptor and VDAC1, through Grp75, that play a role in calcium signaling; (ii) the mitofusins, expressed both on mitochondria and ER membranes, that play a role in tethering and modulating mitochondrial dynamics; (iii) the ER stress sensor PERK that initiates signaling in response to ER stress; and (iv) many more [142–144]. Although it remains unclear how lipids are transported in MAMs, in yeast, it is thought that ER-mitochondrial encounter structure (ERMES) are responsible [145]. ERMES are composed of the structural component maintenance of mitochondrial morphology 1 (MMM1), mitochondrial distribution and morphology 34 (Mdm34), Mdm12, and Mdm10 as well as the regulatory subunit GTPase EF-hand protein of mitochondria (Gem1) [146]. The ERMES complex possesses a synaptotagmin-like mitochondrial lipid-binding (SMP) domain that harbors an elongated hydrophobic groove in which different lipids can bind and possibly be transported [147]. Once the lipids are transported from the ER, mitochondrial enzymes can then synthesize the lipids critical for mitochondrial function.

#### The Interplay Between Mitochondrial Dynamics and Mitophagy

Mitochondrial dynamics and mitophagy have been well studied separately, but investigations aimed at elucidating the interplay between these two components of mitochondrial quality control have been limited. It has been shown that fission can trigger mitophagy and govern mitochondrial clearance [18, 148]. In this regard, multiple studies have demonstrated that alterations to pro-fusion or pro-fission proteins can affect mitophagy, i.e., inhibition of Fis1 in insulin secreting (INS1) cells resulted in a 70% reduction of mitophagy, while overexpression of Drp1 in HeLa cells was accompanied by a 70% decrease in mitochondrial mass [148, 149]. Further evidence that fission and mitophagy are intimately associated is that the Drp1-dependent mediator of fission, endophilin B1, colocalizes with autophagic markers LC3, Atg5, and Atg9 specifically in response to nutrient starvation [150, 151]. Conversely, proteins associated with mitophagy (in particular, excessive PINK1 in depolarized mitochondria) may also play a role in fission by mechanisms that are, at present, unclear. It has been proposed that when PINK1 accumulates and recruits Parkin, Parkin ubiquitinates mitofusins to inhibit fusion [17]. Accordingly, in a state where all mitochondria are depolarized with PINK1 accumulation and mitofusin ubiquitination, the only path for mitochondria would be fission. However, although fission is apparently necessary for mitophagy, mitophagy is not necessary for fission [69, 92, 122, 148, 152, 153].

## Mitochondrial Quality Control in Disease

As discussed in the previous sections, mitochondrial dynamics and mitophagy are essential regulators of mitochondrial quality control and play a role in maintaining mitochondrial homeostasis in healthy cells. Defects in mitochondrial quality control have also been implicated to contribute to both chronic and acute neurological and cardiovascular diseases; however, little is known about how mitochondrial dynamics and mitophagy interact/communicate with each other under pathophysiological conditions.

### Chronic Diseases: Parkinson’s and Alzheimer’s Disease

#### Parkinson’s Disease

The pathologic signature of PD is the accumulation-damaged protein aggregates such as α-synuclein (SNCA) and ubiquitin into intracytoplasmic inclusions termed Lewy bodies. PD has been associated with mutations, sporadic or hereditary, in at least six genes that are responsible for generating mutations in the following proteins: SNCA, Parkin, β-glucocerebrosidase (GBA), PINK1, the protein deglycase DJ1, and leucine-rich repeat kinase 2 (LRRK2) [154]. Interestingly, these genes give rise to proteins that are associated with mitochondria or located within mitochondria, thereby implicating mitochondria as key players in PD [155–158]. Evidence of mitochondrial abnormalities in PC (including reduced complex I activity, reduced mitochondrial membrane potential, increased ROS production, altered mitochondrial dynamics, impaired mitochondrial trafficking, and increases in mtDNA mutations) underscore this association [159–163].

To maintain a healthy mitochondrial network, cells must undergo mitophagy to dispose of damaged and dysfunctional mitochondria and produce new healthy mitochondria via mitochondrial biogenesis. In PD, patients with PINK1 and Parkin mutations display impaired mitophagy. Mutations and defects in PINK1 (i) have the potential to diminish both the mitochondrial translocation and activation of Parkin, (ii) can result in the failure to segregate dysfunctional mitochondria for mitophagy via fission [164], and (iii) have been associated with a decrease in phospho-Drp1 levels and an increase in Drp1 GTPase activity, suggesting a direct role of PINK1 to induce fission [165]. PINK1 deficiency has also been shown to be associated with dysfunctional Na^+^/Ca^2+^ exchangers in the inner mitochondrial membrane that cause unbalanced mitochondrial calcium homeostasis [160]. This impairment of calcium efflux from the mitochondria results in reduced respiration from ROS-stimulated inhibition of glucose uptake. Finally, and not surprisingly based on the aforementioned associations, mutations in PINK1 reportedly increase the sensitivity of cells to stress-induced cell death. Studies have shown that PINK1 is necessary for long-term survival of cells [155, 166].

Mutations in Parkin, on the other hand, can lead to impaired ubiquitination of outer mitochondrial membrane proteins, which has been shown to play a role in recognition by the autophagosome [167]. Interestingly, Parkin was first linked to the mitochondria by evidence that the protein prevented mitochondrial swelling and cytochrome C release in cells treated with ceramide [168]. In addition to this purported neuroprotective role, Parkin was found to protect mtDNA from oxidative stress and stimulate mtDNA repair systems [169], while, in strains of Parkin knockout mice, neurons in the ventral midbrain displayed severe mitochondrial damage and decreases in complexes I and IV, despite being devoid of the phenotypical motor impairment characteristic of PD [170, 171]. It remains unclear how deficiencies in Parkin lead to severe mitochondrial damage and PD. Mitochondria do undergo a basal level of “wear and tear” via mtDNA mutations as well as oxidation of lipids and proteins. As discussed previously, under normal conditions, these damaged mitochondria would be sequestered and undergo proteolytic degradation. If, in PD, defects in PINK1 and Parkin compromise the ability of the cell to degrade and dispose of proteins or damaged mitochondria, the accrual of damaged organelles would, in all likelihood, ultimately lead to cell death.

#### Alzheimer’s Disease

AD currently affects 1.5 million Americans, with the associated memory loss and decline in cognitive function attributed to the accumulation of amyloid-β plaques and phosphorylated tau [172]. Sporadic and hereditary AD are attributed to mutations in several genes, as well as accumulation of mtDNA mutations that generally lead to an increase in β-amyloid levels in the brain [173, 174]. Although the underlying mechanisms are unclear, accumulation of amyloid-β in neurons and formation of plaques have been attributed to excessive cleavage of amyloid precursor protein (APP, a transmembrane glycoprotein) or mutations in the apolipoprotein APOE4, which, under normal conditions, contributes to the breakdown of amyloid-β [175].

[176].

Early in the pathogenesis of AD, mitochondrial abnormalities are also common, including defective glucose metabolism, a reduction in enzyme activity, mitochondrial DNA mutations, defected gene expression, and aberrant mitochondrial dynamics [177]. Mitochondria in AD patients have been observed to reveal significant structural damage together with decreases in mitochondrial fusion proteins, increases in Fis1, and increases in Ser616 phosphorylated Drp1 (despite decreases in total Drp1)—all of which favor excessive fragmentation [178, 179]. In vitro studies corroborated this concept, i.e., overexpression of APP in M17 cells was associated with mitochondrial fragmentation, reduced neurite growth, abnormal mitochondrial distribution, and modulation of mitochondrial fission/fusion proteins [180]. Similar findings were obtained in primary neurons of transgenic mice expressing the human APP Swedish mutation [181]. The interactions between amyloid-β and Drp1 are still unknown, but limited data have proposed that GSK3β may be the mediator in Drp1 phosphorylation via the association of amyloid-β with NMDA receptors and the Wnt signaling pathway [182]. With excessive fission, healthy mitochondria are cleaved unnecessarily, thereby disrupting the equilibration of mitochondrial matrix metabolites (required for efficient production of ATP) and making the mitochondria more vulnerable to injury. In addition, this excessive fragmentation could potentially lead to an upregulation in mitophagic pathways.

#### Insights into I/R Injury

##### Fission, Fusion, and Cell Fate

There has been growing interest in mitochondrial dynamics, and its potential association with apoptosis, in the setting of I/R injury [183–185]. Several studies have uncovered excessive mitochondrial fission or fragmentation during both ischemia and I/R injury [186–189]. Using a 6-h OGD model to simulate ischemia, Kim et al. observed a massive mitochondrial fragmentation profile during OGD in H9C2 cells [186]. This mitochondrial fission profile was confirmed in vivo using a 24-h left anterior descending permanent ligation model in mice [186]. Disatnik et al. and Ong et al. observed mitochondrial fragmentation during reoxygenation in OGD/reoxygenation models using neonatal primary cardiomyocytes and HL1 cells, respectively [188, 189]. In the brain, Tang et al. also demonstrated a highly fragmented mitochondrial profile in mouse N2a neuroblastoma cells following OGD/reoxygenation [187]. Previous studies from our lab, conducted using both primary rat neurons and HT22 cells, revealed evidence of mitochondrial fission during OGD and reoxygenation [190]. Moreover, mitochondrial fragmentation was accompanied by Opa1 processing and concomitant release of cytochrome C [190]. Using an in vitro real-time imaging model of OGD/reoxygenation, we further observed complex temporal alterations in mitochondrial morphology [191]. Using HT22 cells transfected with a plasmid containing a GFP marker, two distinct phases of fragmentation were detected: The first phase of fission occurred during OGD, while reintroduction of oxygen triggered initial fusion followed by complete and massive fragmentation after late reoxygenation. The massive fragmentation observed during late reoxygenation was confirmed in vivo in CA1 hippocampal neurons of rats exposed to global brain ischemia/reperfusion [191].

The aforementioned studies suggest that mitochondrial fragmentation is a pathophysiological consequence of I/R injury and that inhibition mitochondrial fragmentation may reverse this. Indeed, in support of this concept, there is evidence that, after exposure to apoptotic stimuli, Drp1 inhibition or overexpression of a dominant negative Drp1 blocked the induction of apoptosis [192]. Moreover, inhibition of Drp1 was found to be neuroprotective in response to OGD in vitro and transient focal ischemia in vivo [193], and cardioprotective in cultured HL-1 cardiomyocytes subjected to OGD and reoxygenation [194]. However, in the latter study, cardioprotection was only seen when inhibition of Drp1 was initiated as a pretreatment; cell death was paradoxically exacerbated when treatment was administered during reoxygenation [194]. This points to the complexity of mitochondrial dynamics and its effects on cell death or survival—an issue that is highlighted by observations that fusion (presumably favoring survival) involves the formation of lipidic pores that may contribute to mitochondrial permeabilization and compromise cell viability [195], while fission (as discussed above, associated with cell death) is necessary for mitophagy and governs clearance of dysfunctional mitochondria [18, 148]. Thus, despite strong evidence to suggest that mitochondrial fragmentation can be detrimental to the cell during stress conditions, collectively these results reveal a complex dynamic nature of mitochondria that requires further study to understand (i) why fission occurs during these stress states, (ii) why inhibition mitochondrial fission is only cardioprotective when initiated before the OGD/ischemic event, and (iii) why fusion is not possible after reoxygenation/reperfusion.

##### Mitophagy and I/R Injury

I/R injury has been shown to activate mitophagy pathways through multiple signals. During the ischemic phase when ATP production halts, AMPK pathways are upregulated to initiate autophagy [196]. AMPK activates ULK1 via phosphorylation, which will activate the class III PI3K complex (beclin 1, VPS34, and VPS15) that initiate nucleation of the phagophore [197]. Interestingly, ULK1 may have a redundant role in activating mitophagy, i.e., has also been shown to translocate to mitochondria and activate the FUNDC1 receptor [198]. During the reperfusion phase, ROS serves as a signaling molecule to inhibit the mechanistic target of rapamycin (mTOR) pathways, thereby contributing to the initiation and nucleation of the autophagosome [199]. ROS has also been shown to activate mitophagy via BNIP3 although, as stated previously, high levels of BNIP3 can induce apoptosis [200, 201]. Overexpression of BNIP3 in HL-1 myocytes was reported to increase cell death in response to simulated I/R injury by facilitating mPTP opening through the activation of Bcl-2-asscoaited X protein (Bax) [116, 201]. Moreover, BNIP3^−/−^ mice subjected to 1-h coronary artery occlusion and 3-week reperfusion exhibited preserved left ventricular (LV) systolic function and diminished LV dilation, while conditional overexpression of BNIP3 reversed these effects resulting in increased apoptosis and infarct size [202]. Collectively, these results demonstrate a threshold for BNIP3, as increases or overexpression will inevitably lead to increased apoptosis. Given the dual “life-or-death” role of mitochondria, together with reports of the strong association between mitophagic proteins (i.e., BNIP3 and Drp1) and apoptosis, this raises the question of whether mitophagy is beneficial or detrimental to cell fate in response to I/R injury.

#### Heart

During ischemia, upregulation of mitophagy is agreed to confer protection [203, 204]. The most compelling evidence is provided by Kubli et al. Using an in vivo mouse model, the investigators demonstrated that Parkin-deficient mice are more sensitive to myocardial infarction [204]. Following permanent left anterior descending coronary artery occlusion, Parkin-deficient mice displayed accumulation of swollen and dysfunctional mitochondria due to impaired mitophagy, which resulted in larger infarcts and reduced survival rates [204]. Moreover, the investigators observed upregulation of mitophagy with increased expression of Parkin at the border zone of the infarct in wild-type mice [204]. In vitro studies corroborated this concept, i.e., overexpression of Parkin in isolated cardiomyocytes subjected to hypoxia-mediated cell death was associated with increased Parkin translocation to the mitochondria and increased cell viability, while cardiomyocytes expressing Parkinson disease-associated mutants of Parkin failed to reduce hypoxia-mediated cell death [204]. In accordance with this concept, evidence in the in vivo mouse model of permanent coronary ligation revealed that the upregulation of mitophagy via the genetic deletion of two molecular inhibitors, p53 and TP53-induced glycolysis and apoptosis regulator (TIGAR), attenuated apoptotic cell death and provided resistance to subsequent remodeling [203]. Moreover, cardioprotection was reversed in p53^−/−^ and TIGAR^−/−^ mice following permanent myocardial infarction with administration of chloroquine, an autophagy inhibitor, an effect characterized by the accumulation of abnormal mitochondria in the ischemic myocardium [203]. Interestingly, the upregulation of mitophagy via inhibition of p53 and TIGAR was induced through an increase in ROS production followed by BNIP3 activation [203]. In this case, BNIP3 activation was necessary and beneficial in attenuating cardiac I/R injury.

In contrast to ischemic injury, the role of mitophagy in I/R injury remains controversial. A considerable body of evidence suggests that an upregulation of mitophagy during myocardial I/R injury is protective [118, 205–207]. It was first described that upregulation of autophagy in HL-1 cells protected against simulated ischemia-reperfusion by Hamacher-Brady et al. [207]. The investigator’s observations revealed that autophagosomal engulfment of mitochondria was a prominent response in their model. Subsequent studies using HL-1 cells demonstrated an upregulation of BNIP3-regulated mitophagy following simulated I/R [118]. Purportedly, overexpression of BNIP3 during simulated I/R induces mitochondrial damage via ROS production, leading to an upregulation of mitophagy [118]. Expression of ATG5K130R, a dominant negative of ATG5 shown to suppress vacuole formation, significantly reduced mitophagy and increased BNIP3-induced cell death [118]. Together, these data suggests that upregulation of mitophagy occurs following BNIP3-induced mitochondrial damage as a cellular response to remove damaged mitochondria during I/R [118]. More recently, using Langendorff heart I/R model, Lu et al. observed that PGAM5-deficient mice had exacerbated necroptosis in response to 25 min of ischemia followed by 90 min of reperfusion [205]. Data in their mouse embryonic fibroblast (MEF) model of ROS-dependent necroptosis revealed impaired autophagic removal of LC-3II as well as impaired mitochondrial clearance following 24 h of TNF-α cyclohexamide and z-VAD-fmk (TCZ) stimulation. Finally, upregulation of mitophagy has been reported to play a role in the gold standard of cardioprotection, ischemic precondition (IPC) [206]. In both Langendorff perfused rat hearts and in vivo mice subjected to regional IPC, Parkin and P62 translocate to the mitochondria and mediate mitophagy [206]. Moreover, IPC is abolished in Parkin-deficient mice, suggesting a critical role for Parkin in IPC [206]. The investigators propose that selective mitophagy of mitochondria that have the lowest threshold for mPTP opening during IPC would leave behind mitochondria that are more equipped to handle sustained ischemic insults [206].

In contrast to the aforementioned studies, there is some evidence to suggest that the suppression of mitophagy may protect the heart from I/R [208]. In a rat model of left anterior descending coronary artery occlusion, pretreatment with mitochondrial aldehyde dehydrogenase 2 (ALDH2), an allosteric tetrameric enzyme responsible for the metabolism or detoxification of toxic aldehydes, conferred cardioprotection via attenuation of apoptotic cell death [208]. In vitro studies corroborated this concept using H9C2 cells subjected to 2 h of hypoxia (1% oxygen) and 1 h of reoxygenation [208]. Pretreatment of ALDH2 increased cell viability through the suppression of mitophagy [208]. Mitophagy was measured through colocalization of PINK1, Parkin, and the mitochondrial electron transport chain protein cytochrome C oxidase subunit IV (COXIV) [208]. However, given that mitophagy is in constant flux and mitochondria are degraded in autophagolysosomes, mitophagy proteins such as PINK1 and Parkin that are associated with dysfunctional mitochondria would also be degraded. Therefore, evaluation of the mitophagic flux would aid in confirming that ALDH2 is suppressing mitophagy as opposed to enhancing mitophagy and subsequently breakdown of mitophagy proteins.

#### Brain

There are notable differences between brain and heart in that controversy lies with respect to the role of mitophagy in ischemia as well as I/R (beneficial or detrimental). Cerebral ischemic preconditioning has been associated with increased autophagosome formation and confers neuroprotection in rats subjected to permanent middle cerebral artery occlusion (pMCAO) [209]. More recent evidence in the same model of pMCAO confirmed that the upregulation of autophagy during ischemia includes mitophagy, revealed by increased autophagic vacuoles containing mitochondria and LC3 colocalization with COXIV [210]. Interestingly, the upregulation of mitophagy was mediated through Drp1 as treatment with Mdivi, a pharmacological inhibitor of Drp1, prevented mitophagy and resulted in decreased LC3 and COXIV colocalization, increased levels of mitochondrial proteins, and the accumulation of damaged mitochondria following 1 h of pMCAO [210]. Moreover, Drp1 inhibition exacerbated mitochondrial-mediated brain injury to an even greater extent compared to the inhibition of autophagy using 3-methyladenine (3-MA) following 24 h of pMCAO [210]. Conversely, in mice subjected to pMCAO, administration of 3-MA conferred neuroprotection via a reduction in infarct size along with a dose-dependent increase in cell viability following exposure of 4-h OGD in rat primary cortical neurons [211]. Knockdown of Atg7 with siRNA reinforced this concept, resulting in increased cell viability in response to ischemia-induced neuronal injury [210]. Interestingly, the investigators observed no change in infarct volume with administration of mdivi in both in vitro and in vivo models of ischemia [211]. The lack of observable change could be a result of when the inhibitor was administered, i.e., at the point of artery occlusion or the beginning of OGD. Results from our lab demonstrate that mitochondria undergo massive fragmentation within 20 min of ischemia [191]. In this case, mitochondrial fragmentation could have occurred before the onset of inhibition with mdivi, resulting in segregated mitochondria ready to be recruited for mitophagy.

In cerebral I/R, there is a consequential amount of literature that suggests that an upregulation of mitophagy confers neuroprotection [205, 211, 212]. The strongest evidence to support this concept is provided by Zhang et al. [211]. Using pharmacological and genetic suppression of autophagy (i.e., 3-MA treatment, bafilomycin A1 (BafA) treatment, Atg5 knockout, and Atg7 knockdown), primary cortical neurons subjected to 2 h of OGD followed by a 24-h reperfusion had significantly decreased cell viability as compared to controls [211]. These results were affirmed in an in vivo transient MCAO model, where inhibition of autophagy via 3-MA treatment and Atg7 knockdown exacerbated I/R injury [211]. Moreover, addition of rapamycin, a known enhancer of mitophagy that attenuates mitochondrial dysfunction following cerebral ischemia [212], partly reversed the deleterious effects of 3-MA-treated primary neurons subjected to OGD reperfusion [211]. Similar to the heart, PGAM^−/−^ mice subject to transient MCAO displayed significantly higher infarct sizes as compared to wild-type mice after 72 h of reperfusion [205].

At variance with the preceding studies, evidence provided by Shi et al. suggests that excessive mitophagy following cerebral I/R results in cell death [213]. Using an adapted Rice-Vanucci model of neonatal stroke or hypoxia-ischemia encephalopathy (HIE), the investigators observed that pups deficient of BNIP3 had a decrease in mitophagy that resulted in significantly smaller infarct sizes in response to neonatal stroke and 7 days of reperfusion [213]. Interestingly, the infarct volume in BNIP3^−/−^ pups was significantly larger after 1 day of reperfusion but then recovered while the infarct volume in wild-type pups was exacerbated after 3 to 7 days of reperfusion [213]. The investigators also demonstrated in wild-type pups a dramatic increase in BNIP3 mitochondrial-localized homodimer expression in a time-dependent manner following neonatal stroke accompanied by a significant decrease in mitochondrial proteins from isolated cortical neurons following 6 h of OGD [213]. Although these data were collected in a neonatal model, it suggests that (i) BNIP3 induces excessive mitophagy following I/R, which ultimately exacerbates cerebral I/R injury, and (ii) underscores the balance of mitophagy required to prevent pro-apoptotic proteins such as BNIP3 to surpass the “death signal” threshold.

### Beyond “Good” Versus “Evil”: a Question of Balance?

**Fig. 5:**
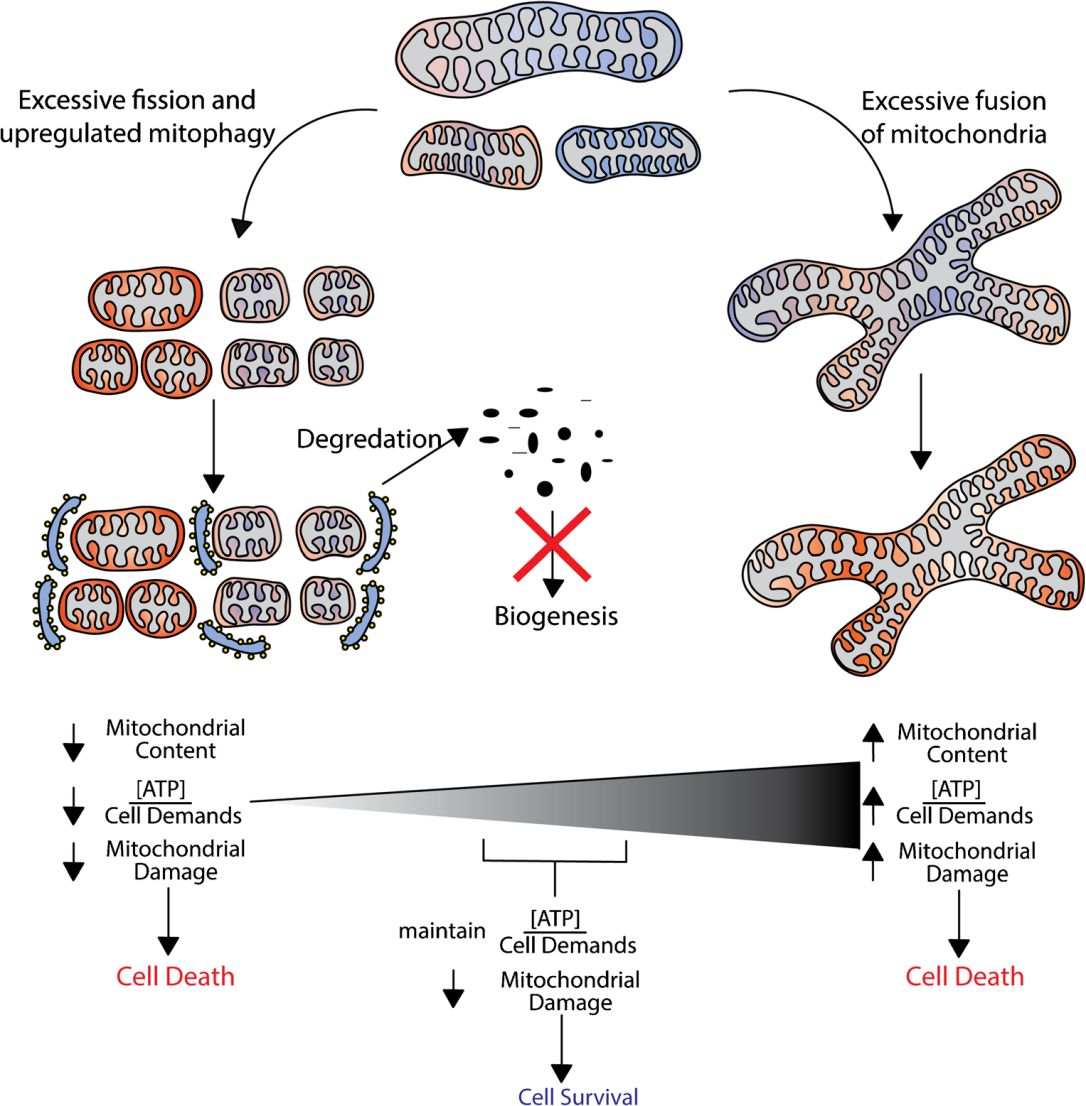
Finding a balance of mitochondrial quality control. During I/R injury, there is excessive mitochondrial fragmentation, favoring an increase in mitophagy. Degradation of dysfunctional ROS-producing mitochondria is critical for survival; however, mitochondrial content decrease would compromise ATP production. Insufficient ATP production paired with inhibited biogenesis will ultimately lead to cell death. In contrast, if fission was completely inhibited, mitochondrial content would be maintained, but damaged mitochondria would not be segregated and could lead to accumulated mitochondrial dysfunction and exacerbate damage to the entire mitochondrial network. The increase in mitochondrial damage would augment pro-apoptotic stimuli and, ultimately, cause cell death. Therefore, a balance in mitochondrial quality control (i.e., an equilibrium between retaining adequate mitochondrial content for sufficient ATP production versus disposal of dysfunctional mitochondria) is optimal for cell survival after I/R injury

As reviewed in the preceding sections, there is continued controversy regarding whether upregulation of mitophagy in the setting of I/R is good or bad. It may, however, be more appropriate to consider mitophagy (and mitochondrial quality control as a whole) as a balancing act (Fig. 5). For example, it has been proposed that, following reperfusion, mitophagy is essential to clear dysfunctional mitochondria [211]. However, excessive mitophagy coupled with inhibited mitochondrial biogenesis and a global decrease in protein synthesis [214, 215] will result in a decrease in mitochondrial mass and, subsequently, a deficit in ATP production that may fail to meet the demands of the cell. This energy imbalance could eventually cause energy deprivation and cell death. With the other extreme—that is, with minimal mitophagy—damaged mitochondria will not be eliminated and the overall ROS burden would increase. This excessive ROS formation could further induce mitochondrial dysfunction, leading to a feed forward cycle of ROS production and ultimately cell death (Fig. 5).

Accordingly, there is a fundamental need for balance in mitochondrial quality control, and further investigation is needed to define this threshold. If this threshold can be identified, modulation of mitophagy may represent a valuable therapeutic option, with the goal of eliminating dysfunctional mitochondria while still providing sufficient energy to repair cellular damage, restore protein translation, and ultimately return to homeostasis. PINK1 and Parkin (rather than BNIP3 and NIX) may yield the greatest promise as effective targets for manipulation of mitophagy, given their reported favorable association with cell survival together with a lack of involvement in apoptotic pathways [204, 206, 211].

## Conclusions and Challenges

Mitochondrial quality control is critical for the homeostasis of the mitochondrial network, and a constant balance is needed between mitochondrial fission/fusion as well as mitophagy and biogenesis. Disruption of mitochondrial quality control has been proposed to contribute to the pathogenesis of acute and chronic diseases, including Parkinson’s disease, Alzheimer’s disease, and ischemia-reperfusion-induced cell death in the brain and heart. Accordingly, targeted modulation of one or more of the molecular components involved in mitochondrial quality control provides opportunities for the design of novel therapies. However, to capitalize on this potential opportunity, a greater mechanistic understanding of mitochondrial fission/fusion, mitophagy, and mitochondrial quality control—together with the development improved molecular tools to investigate these complex phenomena [216]—will be required.
